# Using computed tomography to recover hidden medieval fragments beneath early modern leather bindings, first results

**DOI:** 10.1186/s40494-023-00912-9

**Published:** 2023-04-24

**Authors:** J. Eric Ensley, Katherine H. Tachau, Susan A. Walsh, Honghai Zhang, Giselle Simon, Laura Moser, Jarron Atha, Paul Dilley, Eric A. Hoffman, Milan Sonka

**Affiliations:** 1 Special Collections & Archives, University of Iowa Libraries, Iowa City, IA 52242 USA; 2 Department of History, University of Iowa, Iowa City, IA 52242 USA; 3 Small Animal Imaging Core, Iowa Institute for Biomedical Imaging, University of Iowa, Iowa City, IA 52242 USA; 4 College of Engineering, University of Iowa, Iowa City, IA 52242 USA; 5 Department of Conservation and Collections Care, University of Iowa Libraries, Iowa City, IA 52242 USA; 6 Department of Classics, University of Iowa, Iowa City, IA 52242 USA; 7 Department of Radiology, Carver College of Medicine, University of Iowa, Iowa City, IA 52242 USA; 8 Department of Religious Studies, University of Iowa, Iowa City, IA 52242 USA; 9 Iowa Institute for Biomedical Imaging, University of Iowa, Iowa City, IA 52242 USA; 10 Visualization Lab, Iowa Institute for Biomedical Imaging, University of Iowa, Iowa City, IA 52242 USA

**Keywords:** Medieval manuscripts, CT scanning, Fragments, Conrad Gessner, Early Printed Books

## Abstract

Medieval bindings fragments have become increasingly interesting to Humanities researchers as sources for the textual and material history of medieval Europeans. Later book binders used these discarded and repurposed pieces of earlier medieval manuscripts to reinforce the structures of other manuscripts and printed books. That many of these fragments are contained within and obscured by decorative bindings that cannot be dismantled ethically has limited their discovery and description. Although previous attempts to recover these texts using IRT and MA-XRF scanning have been successful, the extensive time required to scan a single book, and the need to modify or create specialized IRT or MA-XRF equipment for this method are drawbacks. Our research proposes and tests the capabilities of medical CT scanning technologies (commonly available at research university medical schools) for making visible and legible these fragments hidden under leather bindings. Our research team identified three sixteenth-century printed codices in our university libraries that were evidently bound in tawed leather by one workshop. The damaged cover of one of these three had revealed medieval manuscript fragments on the book spine; this codex served as a control for testing the other two volumes to see if they, too, contain fragments. The use of a medical CT scanner proved successful in visualizing interior book-spine structures and some letterforms, but not all of the text was made visible. The partial success of CT-scanning points to the value of further experimentation, given the relatively wide availability of medical imaging technologies, with their potential for short, non-destructive, 3D imaging times.

## Introduction

The advent of the European printing press in the mid-fifteenth century resulted in a remarkable expansion of the annual production of books, with a consequent increase in the work of bookbinders. In university towns, capital cities, and other urban centers where book cultures flourished, binders’ workshops were already well established before the first printed codices arrived in their hands; the fact that the gatherings of a book had been impressed mechanically rather than written by hand did not, in itself, affect the methods used in any workshop for binding books [1]. What did change, however, was which particular manuscripts contemporaneous readers decided had been superseded by the relative “standardization” of the manuscript’s text upon being printed in multiple copies. Once rendered obsolete, a parchment manuscript was especially likely to be sold and reused in other ways, including by being dismembered and cut into parchment fragments (“binders’ waste”) useful for strengthening parts of a binding [2, 3].

Already in the sixteenth century, scholars and antiquarians were collecting manuscript fragments; by the seventeenth century, paleographers and bibliophiles were gathering and studying them; and by the eighteenth, antiquarians were retrieving parchment fragments from early-modern bindings [4–7]. Contemporary study of medieval manuscript fragments (or the “*membra disiecta*” of ancient and medieval writings) was propelled immeasurably by the research of Neil R. Ker [8–10]^1^ and Shelomo Goitein’s reconstruction of medieval life around the Mediterranean from the dispersed troves of written fragments from the Cairo Geniza [11–13]. (Although the estimated 400,000 Cairo Geniza fragments come primarily from correspondence rather than books, they share many of the problems and possibilities that book fragments pose for scholars’ investigations.) These and subsequent researchers have demonstrated that manuscript fragments, both singly and when available in sufficient numbers to yield quantifiable data, contribute meaningfully to our understanding of the past. Increasingly, rare book librarians, book conservators, codicologists, and historians have come to appreciate that the recycling of dismembered manuscripts in bindings has “served as an inadvertent mechanism for textual preservation” rather than solely as evidence of destruction [14, 15]. Not surprisingly, therefore, the new interdisciplinary field of “Fragmentology” is burgeoning.

Thanks to fragment collectors from the sixteenth century onwards, most major library collections of ancient and medieval manuscripts possess such *membra disiecta*. Many had been separated from the binding structures that they once supported by collectors up through the early twentieth century, who replaced (or simply opened) intact bindings that they deemed “ordinary” or outdated, far more readily than responsible librarians, conservators, and scholars would countenance today. Indeed, numerous medieval and early-modern bindings are themselves works of art in their own right. Yet, as medieval parchment pastedowns or scraps visible under deteriorated or dismembered bindings show, their late-medieval and early-modern makers often included medieval materials to reinforce binding structures. In light of that common practice, scholars know that many portions of medieval books remain undiscovered within intact bindings. If, therefore, we and future medievalists are to find and visualize such medieval materials, non-destructive methods will be required.

Since 2011 several publications have detailed experiments by researchers in Rome who have applied non-invasive, non-destructive Infrared Thermography (IRT) to detect and visualize hidden medieval manuscript fragments glued between the outer boards and translucent inner endleaves of early modern book bindings [16–21]. The experimental samples include thirteenth- through eighteenth-century books with parchment bindings. The research team’s IR camera recorded series of thermograms from artificially induced temperature changes in these books through two active methods requiring different configurations of lamps: flash lamps for pulsed IRT and halogen lamps for lock-in IRT. Both methods depend upon the semi-transparency of the parchment or paper covering the hidden fragments and are limited in the depth below the surface to and from which IR radiation travels (in these studies, 0.1 mm). In combination, pulsed and lock-in IRT revealed the binding structures and visualized the writing on manuscript fragments beneath several bindings’ endleaves, although developing a process to correct for edge distortion for improved legibility of detected writing remains ongoing [18–22].

In 2017, Jorien Duivenvoorden, Anna Käyhkö, Erik Kwakkel, and Joris Dik, who estimate that as many as twenty percent of early modern books contain medieval parchment scraps or larger fragments, published the results of the first experiment of which we are aware that employs macro X-ray fluorescence (MA-XRF), a second non-invasive, non-destructive means to visualize such hidden medieval materials within intact bindings [24]. Using a Bruker M6 Jetstream mobile MA-XRF scanner, Duivenvoorden and his colleagues successfully visualized texts hidden in or on the spines of three books, printed in 1555, 1567, and 1632, that are held by the Leiden University Libraries.^2^ The first of these books is covered by a repurposed manuscript fragment, its text mostly obscured under a layer of black paint; in the second, a paper pastedown obscures the text of an underlying fifteenth-century manuscript fragment on the inside cover; in the third, a thin medieval parchment strip with text reinforces the spine underneath a limp parchment cover (a typical early modern binding). The MA-XRF technology made possible the visualization and reading of the texts beneath the paint, paper, and parchment layers of these three books by the investigative team. They found, however, that the MA-XRF techniques did not recover any text in a fourth sample with a leather binding, which the X-rays did not penetrate. Duivenvoorden et al*.* also encountered two further problems in their efforts to obtain legible high-resolution images. First was a problem of focus, because movement of the scanning head containing the X-ray source and detector, which traveled on a plane, could not be adjusted automatically during the scan to keep the same precise distance from each millimeter of the scanned book’s curved spines, with all their normal, variable irregularities. Any parchment fragments within the spine will also have idiosyncratic curvatures and anomalies. Second, the Leiden investigators discovered that use of the mobile MA-XRF technology requires a long scanning time — six to sixty-two hours — which renders its use impractical for systematically scanning bindings to discover and visualize hidden fragments within them [24].

The research challenges of imaging book spines that Duivenvoorden et al*.* faced are among those that researchers at the University of Iowa’s “Iowa Initiative for Scientific Imaging and Conservation of Cultural Artifacts” (IISICCA) are seeking to resolve. Founded in 2021 by an interdisciplinary team of University of Iowa faculty and staff specialists from the departments of Art History, Classics, History, Religious Studies, and Engineering, the Center for the Book (UICB), the Stanley Museum of Art, the University Libraries, the Iowa Institute for Biomedical Imaging (IIBI), Small Animal Imaging Core (SAIC), and the Iowa Initiative for Artificial Intelligence (IIAI), IISICCA’s collaborators apply enhanced non-destructive digital imaging, machine learning, and artificial intelligence (AI) technologies to the study of cultural artifacts, including books and manuscripts. IISICCA’s members appreciate that IRT and mobile MA-XRF technologies may permit the imaging in situ of any rare books that are too fragile to be moved, although published studies do not indicate whether this has actually been attempted. Nevertheless, both technologies require the purchase or customization of expensive equipment and software for which experience in calibration, use, and interpretation must then be developed. For many libraries and researchers in the humanities, this is not financially feasible.

IISICCA researchers hypothesized that the difficulties that the Leiden researchers had encountered could be solved by replacing MA-XRF imaging methods with computed tomography (CT) imaging, which acquires data far more quickly than can mobile MA-XRF and, by producing 3D rather than 2D images, avoids the latter’s problems of focus. IISICCA collaborators therefore decided to experiment with medical CT technologies, because campuses with medical schools usually already possess or have access to medical CT imaging nearby. CT scanners designed for clinical use offer several benefits: first, they must be approved by the FDA (or comparable agency elsewhere), which means that the X-ray dosage for each scan is known; such CT scanners have daily quality control workflows; the machine must be operated by a board-certified individual, who is trained to use the proprietary CT scanner software. These elements aid in the repeatability of experiments, and provide the foundation for quantitation across data sets. Having this control enables researchers to be conscious of protecting the cultural heritage objects from unnecessary radiation doses. The absorbed radiation dosages produced by CT scanners designed for human subjects are significantly lower than the doses of absorbed radiation employed in the most recent research to confirm that X-ray irradiation is non-destructive of aged parchment and paper [23]. Finally, the large gantry of a medical CT scanner permits the scanning of larger objects and, as these scanners have beds that move through the gantry, multiple cultural objects can be imaged in one scanning workflow. All scanning methods require the expertise and direction of book conservators to prevent harm to the cultural heritage in their safekeeping. The chief disadvantage of medical CT scanning is that it is more time-consuming for the book conservator to prepare and stabilize books for transport than are in situ methods.

## Previous Use of Computed Tomography to Recover Inaccessible Texts in Books

The most basic use of CT scanning to study manuscripts has been focused on determining the structure of a written object, especially one which is too fragile or damaged to be opened safely for examination [25]. The resulting images can be quite useful for understanding the physical structure of the manuscript. The captured data can also be visualized using computer algorithms to identify page structure in codices, or the folding path of a scroll, and to digitally “flatten” 3D images to 2D more akin to the experience of viewing an open, or unfolded, manuscript — a procedure of “virtual unrolling” pioneered by Brent Seales and his team on ancient scrolls [26–29]. The same technology and methods of imaging and processing were later applied to an early Coptic manuscript of the Acts of the Apostles at the Morgan Library, M910 [30]. Recently, a technique of “virtual unfolding” has been employed with CT-scanned papyrus samples, in one of which a Coptic word has been recovered [31]. CT scanning has been used in combination with other imaging technologies, such as Multispectral Imaging (MSI), in an attempt to read text hidden within mummy cartonnage, with some promise but not yet with full success [32]. Being able to virtually unroll or unfold an object does not guarantee that its hidden text will suddenly be legible or even visible. The En Gedi scroll and Acts of the Apostles codex, both made of parchment, feature iron gall ink which is visible, at least in part, after image post-processing. The ink of the Herculaneum papyri, in contrast, is made of carbon, and has proven more difficult to read, although there are grounds for optimism and efforts are ongoing [33, 34].

## Our Experiments with CT scanning

IISICCA members, too, have prior experience with CT imaging to visualize the codex within a small (57 × 57 × 20 mm) sealed, leather-bound talisman, and established that we could visualize a legible text within a similar book that no longer had its leather cover [35]. IISICCA includes and relies upon the expertise of both a book conservator and a curator of rare manuscripts to select only appropriate materials for imaging, to establish that scanning methods are indeed non-destructive, to follow approved conservation protocols in preparing for transport across campus and the concommitant changes in temperature and humidity, and to stabilize the materials to be scanned on the CT scanner’s movable table. For the present experiment, we also decided to apply image processing methods developed in the medical sciences.

**Fig. 1:**
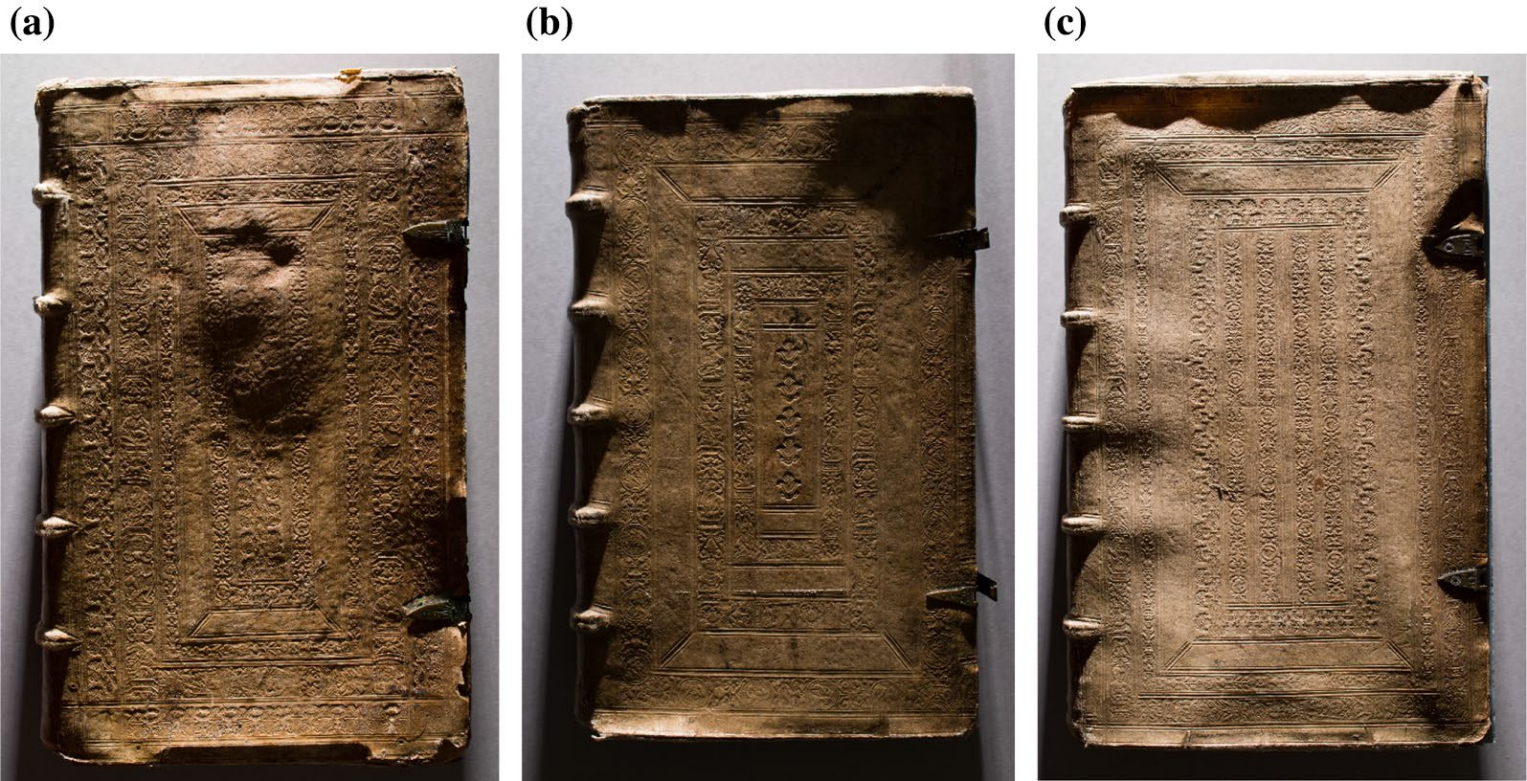
Conrad Gessner’s five-volume *Historia animalium*, front covers, tooled pig-skin, raking light. University of Iowa Libraries Special Collections and Archives, x-Collection Super FOLIO QL41.G37. Photos: Emma Guerard. **a**: tome 1, containing vol. 1 **b**: tome 2, containing vols. 2–3. **c**: tome 3, containing vols. 4–5

**Fig. 2:**
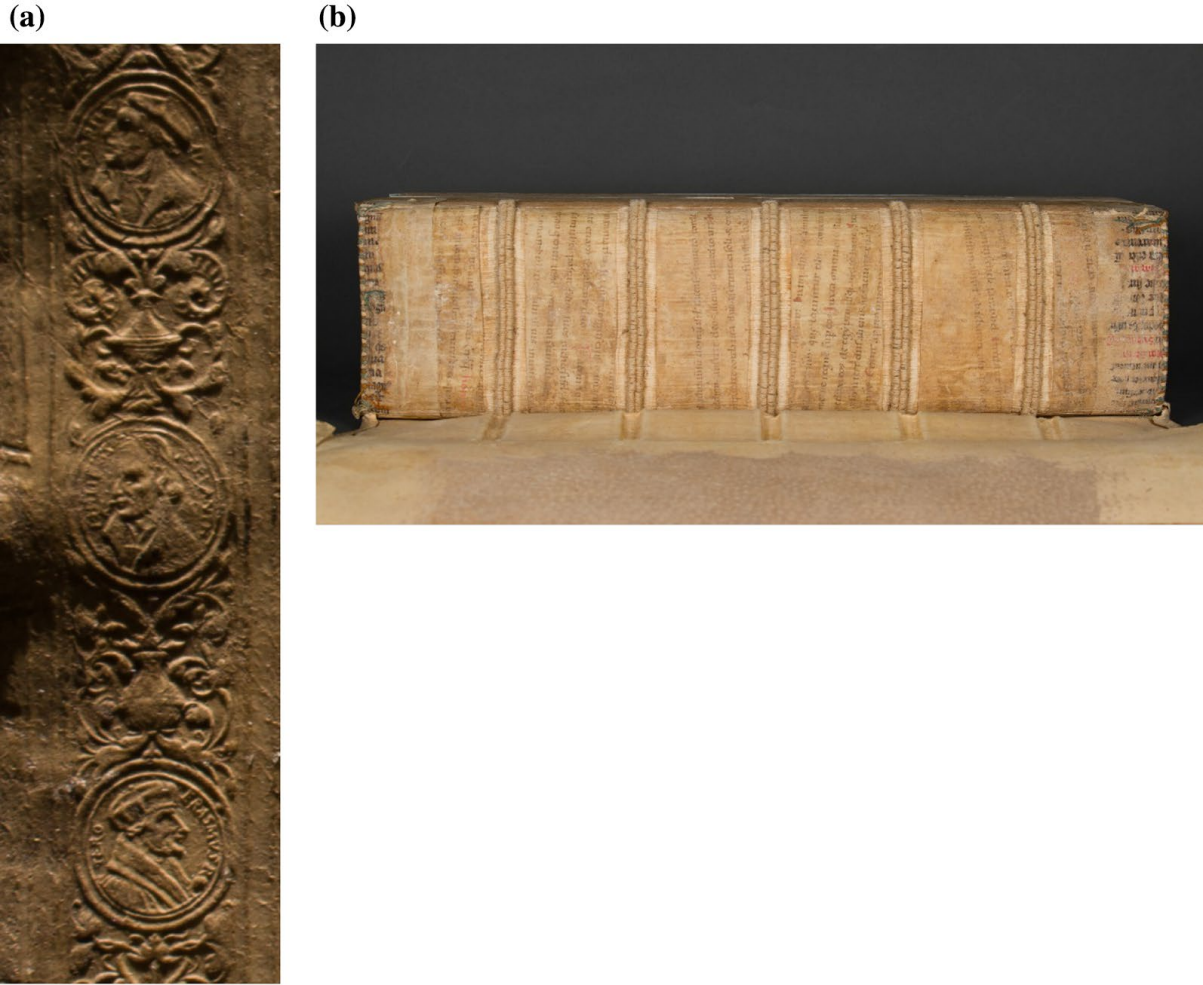
**a** Detail of 1b; medallions from top to bottom: Martin Lu(ther), Philippus Melancthon, Erasmus Rotero(damus). **b**: Tome 3 (as in 1c above) of Conrad Gessner’s *Historia animalium,* open spine of control book revealing medieval binding fragments. Photo: Emma Guerard

Our IISICCA team found an excellent test case in The University of Iowa Libraries Special Collections and Archives’ complete copy of Conrad Gessner’s five-volume *Historia animalium* (1551–1587, printed in Zurich). One of the earliest printed attempts at a holistic encyclopedia of the world’s fauna, the volumes at The University of Iowa are consolidated into three tomes, each with an elaborately stamped, tawed pig-skin binding, emblematic of the style sometimes found in Germany in the sixteenth century (Fig. 1 a-c). To judge from the three bindings’ shared decorative style, and use of at least one stamp or roll on two of the three volumes, this set of the *Historia animalium* was evidently bound in the same workshop. The binding of one tome has on the front (in the outermost rolled, decorated rectangular panel) a repeated trio of portrait medallions, which contain the names *Martin Lu(ther)*, *Erasmus Rotero(damus)*, *Philippus Melancthon* (Fig. 2a); although their works were read in many regions of sixteenth-century Europe, as an ensemble their presence was most resonant in German-speaking lands. Hence, their stamped representations offer further support for the identification of the bindings as the work of a German workshop.

**Fig. 3:**
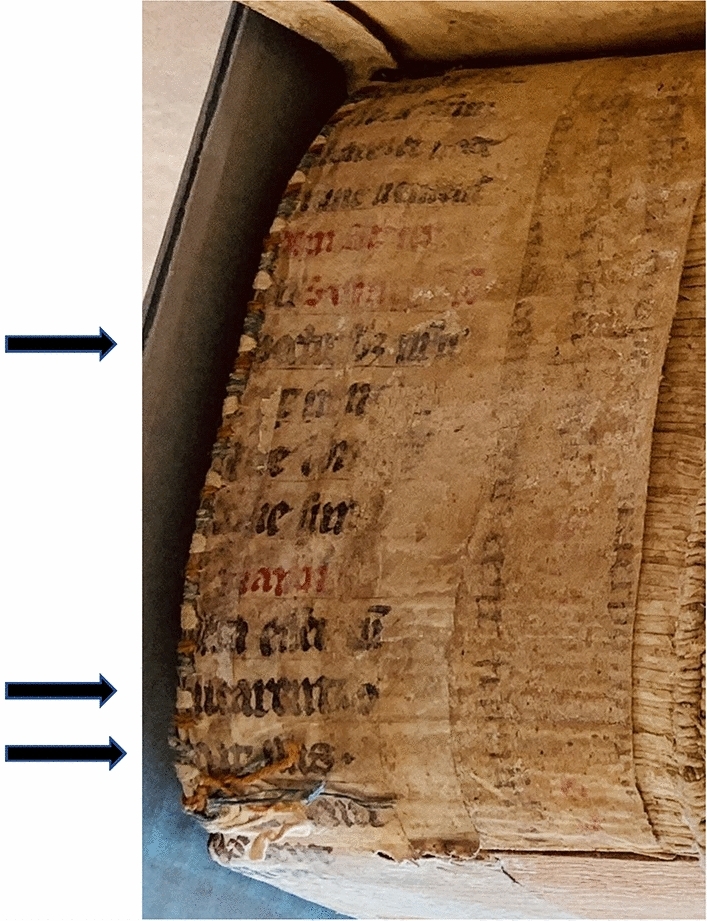
Detail of 2b (Tome 3 of Conrad Gessner’s five-volume *Historia animalium*, containing vols. 4–5, exposed spine), parchment strip at tail, with endband stitching in blue-green, red, and white threads, with stitches through individual letters as indicated by arrows

What made these three codices apt candidates for our experiments is the fact that one of them has a damaged binding, which exposes its spine and sewings when retracted (Fig. 2b). Reinforcing the spine between the sewing stations and at the book’s head and tail are several substantial medieval parchment fragments with Latin text. Such pasted parchment “transverse spine liners” (known to some codicologists as “patch spine liners”), which extend horizontally across the width of the spine between the five sewing stations, “are intended to help to stabilize the text-block and control the operation of the spine in terms of its opening characteristics” [1]**.** Additional parchment layers visible at the head and tail must have been glued or pasted to the spine before the sewing of endbands, as is clear from stitches that bifurcate several letters (Fig. 3). The script on these end strips is a (probably thirteenth-century) Gothic bookhand, written primarily in dark black ink with what appear to be vermilion rubrics (Lat. *rubeus*, red). The remaining six transverse-liner fragments derive from an earlier manuscript of the Latin Bible, written in clearly legible proto-Gothic hands of the late eleventh or early twelfth century. Interspersing the now light brown (probably iron-gall) ink of these six fragments, all from 1 Kgs (1 Sam) chapters 7–8,^3^ are red letters at the start of verses.

The exposure of the spine of one of a three-volume set not only gave us reason to think it probable that the artisans who bound one employed the same procedures for all three — in which case the two intact bound spines should likewise contain parchment binders’ waste — but also gave us a means of checking whether the CT scan was capturing all the visible layers of parchment and the discernible variety of inks because the binding could be retracted and put back in place on the damaged volume In other words, the tome with the damaged binding became our control.

## Methods and Results

To look for the presence and investigate the contents of parchment spine linings, all three Gessner *Historia animalium* codices were imaged using a medical, dual-source, dual-energy (DECT) scanner (Siemens SOMATOM Force CT). This scanner incorporates advances that allow ultra-low dose (down to 0.15 mGy) imaging [36] and significantly higher spatial and contrast resolution. By using two X-ray imaging chains including high power X-ray tubes (which provide high mAs and kV settings from 70–150) spiral scanning is accomplished with rapid table motion, enabling volumetric acquisitions in under a second when using information from both imaging chains in the reconstruction. The high power X-ray tubes also allow for tin filtering to narrow the X-ray spectrum. Spiral scanning at table speeds of up to 737 mm/sec can be accomplished while maintaining good spatial resolution, because there are effectively 384 rows of detectors (192 × 2) when both imaging chains are used for one reconstruction. Our equipment’s spatial resolution can be up to 32-line pairs per cm with a voxel size down to 0.24 mm. The X-ray imaging chain pair can be rotated at a speed of up to 0.25 rotations per second. The increased sensitivity of the scanner’s Stellar Infinity detectors facilitates its low dose imaging, while Siemens’ “advanced modeled iterative reconstruction” method (Admire) reduces the inherent noise associated with low dose scanning [37, 38]. Future imaging will take advantage of the dual energy capabilities to customize kV settings to explore the differentiation of inks based upon their compositions. However, in the present study, concentrating on maximum spatial resolution and optimization of image contrast, we used the protocol parameters shown in Table 1.

**Table 1:** Ultra-high resolution (UHR)

Focal spot size: 0.4 kVp: 70 mAs: 200 Pitch: 0.85 (an index of table speed relative to gantry rotation speed) Rotation time: 1 s Reconstruction slicing: 0.4 × 0.2 mm (slice thickness and slice increment) Kernel: Uq69, ADMIRE at a strength of 5	

^*^This is the technical description provided by the maker of the DECT scanner used

**Fig. 4:**
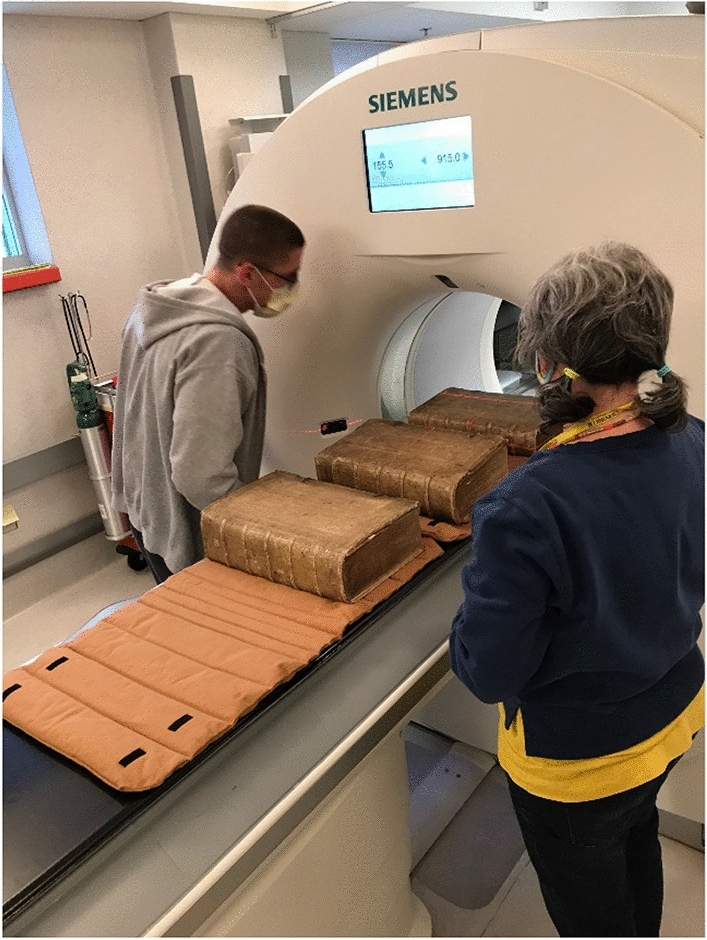
Placement of the three Gessner volumes on conservator-approved library cushions. Laser alignment positioning on the mobilized table in the gantry of the DECT scanner. Photo: Laura Moser

During initial positioning, the books are stabilized and placed on the mobilized table, which slides through the scanner gantry. During the acquisition, the X-ray source and detectors rotate around the immobilized books as the table moves through the gantry. After a few minutes of setup for all three tomes, scanning the full depth of the book spines required five seconds per spine (Fig. 4) rather than the 6 to 66 h required for mobile MA-XRF scanning in Leiden. In this study, the scan of each book was sectioned into 8 reconstructed 3D image volumes to reduce the in-plane voxel dimensions to below the innate scanner resolution (below 0.25 mm). These 8 image sets were defined by the top and bottom half of each book along with the 6 segments (top to bottom of the book) defined by the endbands and the raised bands on the spines. For each book the stack of slices ranged from 343 to 520 chosen to incorporate the complete structure of each book’s spine. The field of view for the top and bottom half of each book was 21.5 cm and the fields of view of the spinal segments (6 per book) were 8.5, 11.0 and 12.0 cm respectively. Thus, with a reconstruction matrix of 512 × 512 voxels, a voxel size for the six spinal segments of a given book ranged from 0.17 × 0.17 × 0.2 mm to 0.23 × 0.23 × 0.20 mm.

**Fig. 5:**
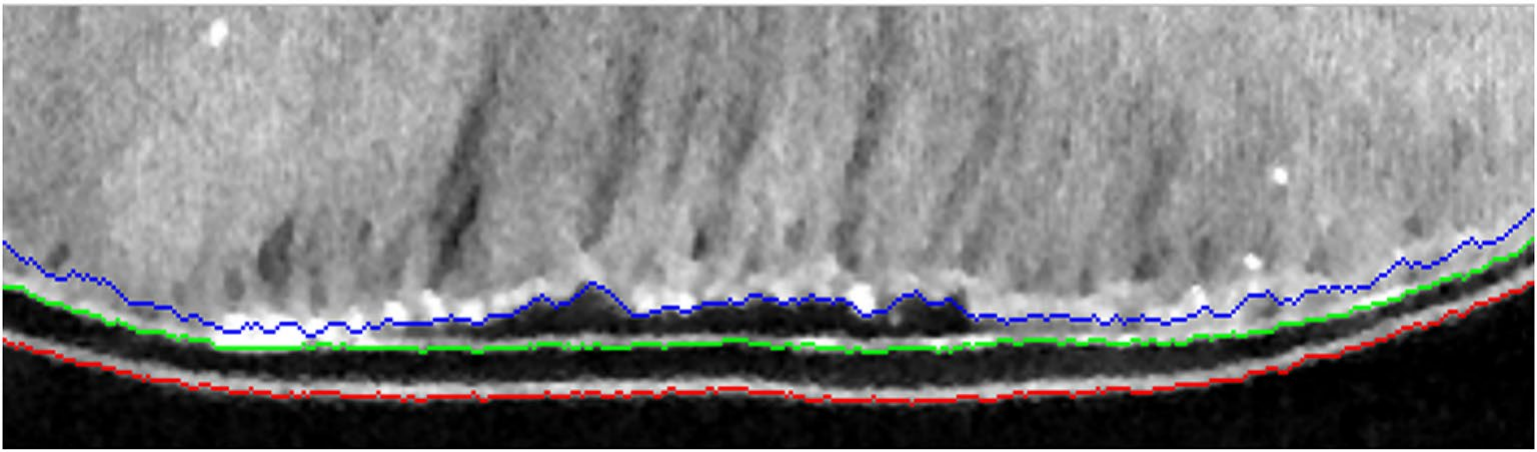
In this detailed view, one slice of the volumetric 3D CT image acquired of one volume is shown, in which the spine’s three layer surfaces have been segmented by LOGISMOS. The spine is formed of the gatherings of folded paper sheets of the book’s textblock (blue); parchment spine patches placed on the spine (green); and tawed pigskin cover (red)

**Fig. 6:**
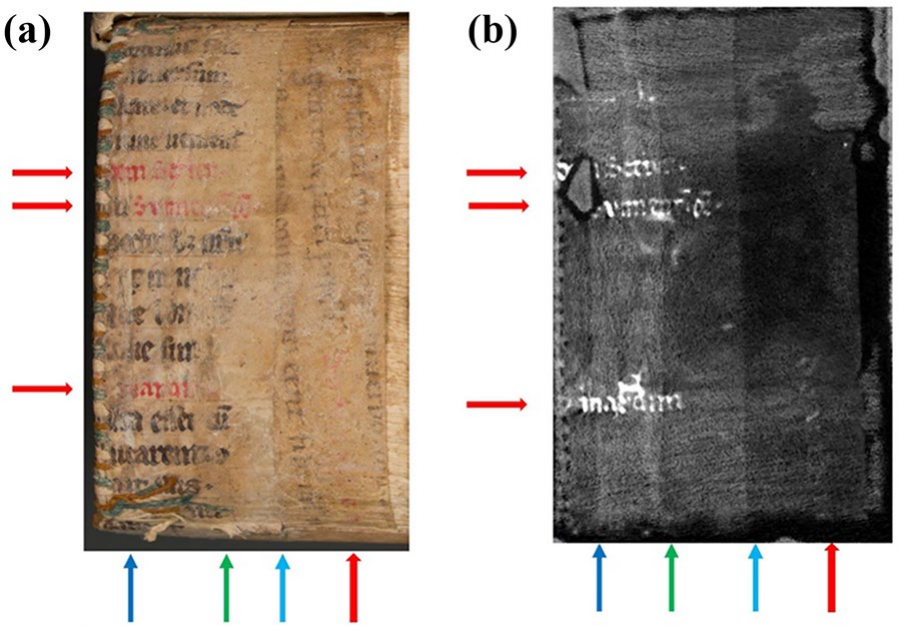
(detail of Fig. 2b). Comparison of parchment images taken by digital camera (a) and CT scan processed with LOGISMOS (b). In both (a) and (b), the horizontal and vertical red arrows point to the location of red lettering; in (b), the dark blue line points to the edge of the underturned portion of the first strip, the green line points to the edge of the second strip where it extends underneath the first, and the turquoise blue line points to the right edge of the first strip

**Fig. 7:**
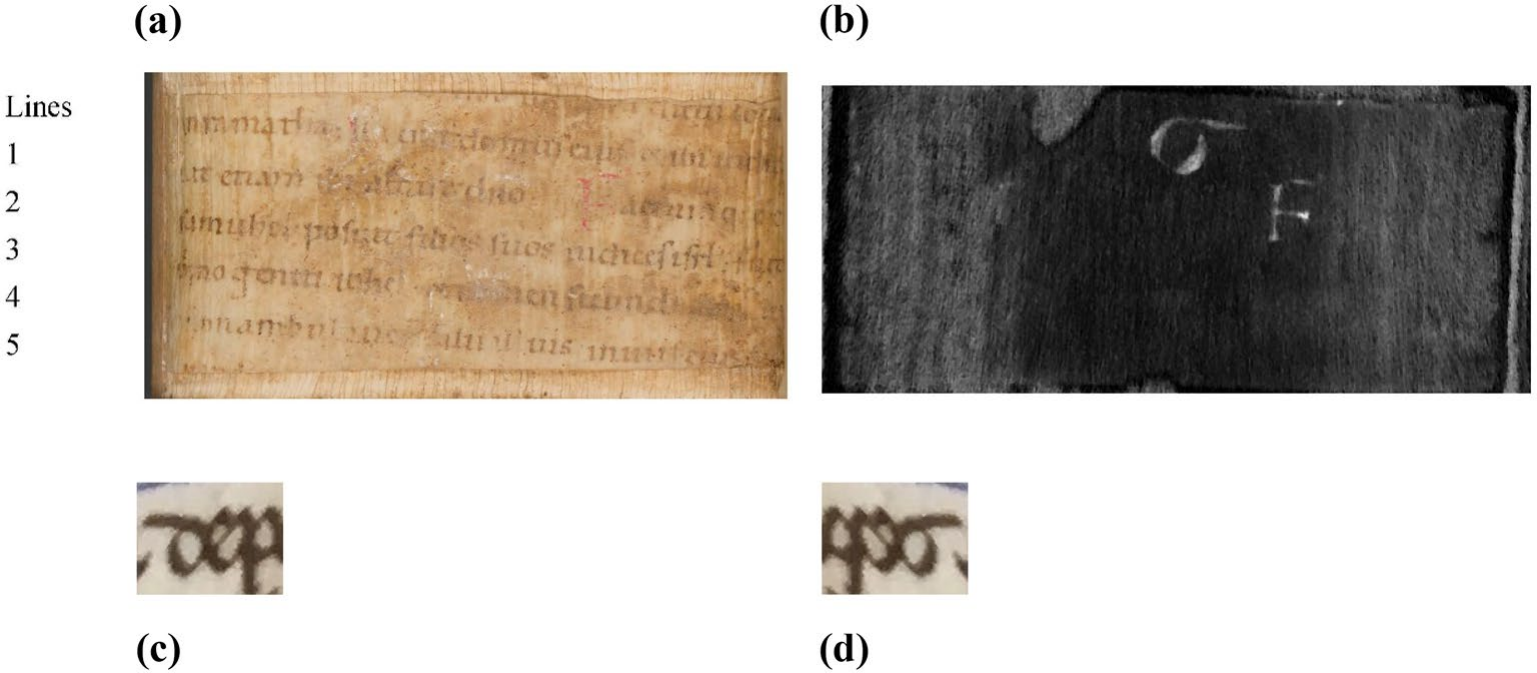
Comparison of images of a transverse parchment patch from the control volume as taken by digital camera (**a**) and acquired by CT, processed by LOGISMOS (**b**). Figure 7a is a detail of Fig. 2b above, showing the transverse patch between the first and second stitching stations on the Left. Note that the second line of (**a**) contains a faint red majuscule ***F*** that is more clearly visible in (**b**), and an additional letter of roughly the same size in (**b**) that is not visible in (**a**). An example (**c**) of a lowercase uncial letter ***D*** (in a word beginning “dep-”), ca. 1195–1210 CE from Vienna ÖNB Cod. 2554, fol. 1r, lin. 1; the same example reversed (**d**). Digital photo of Vienna manuscript folio: Katherine Tachau

**Fig. 8:**
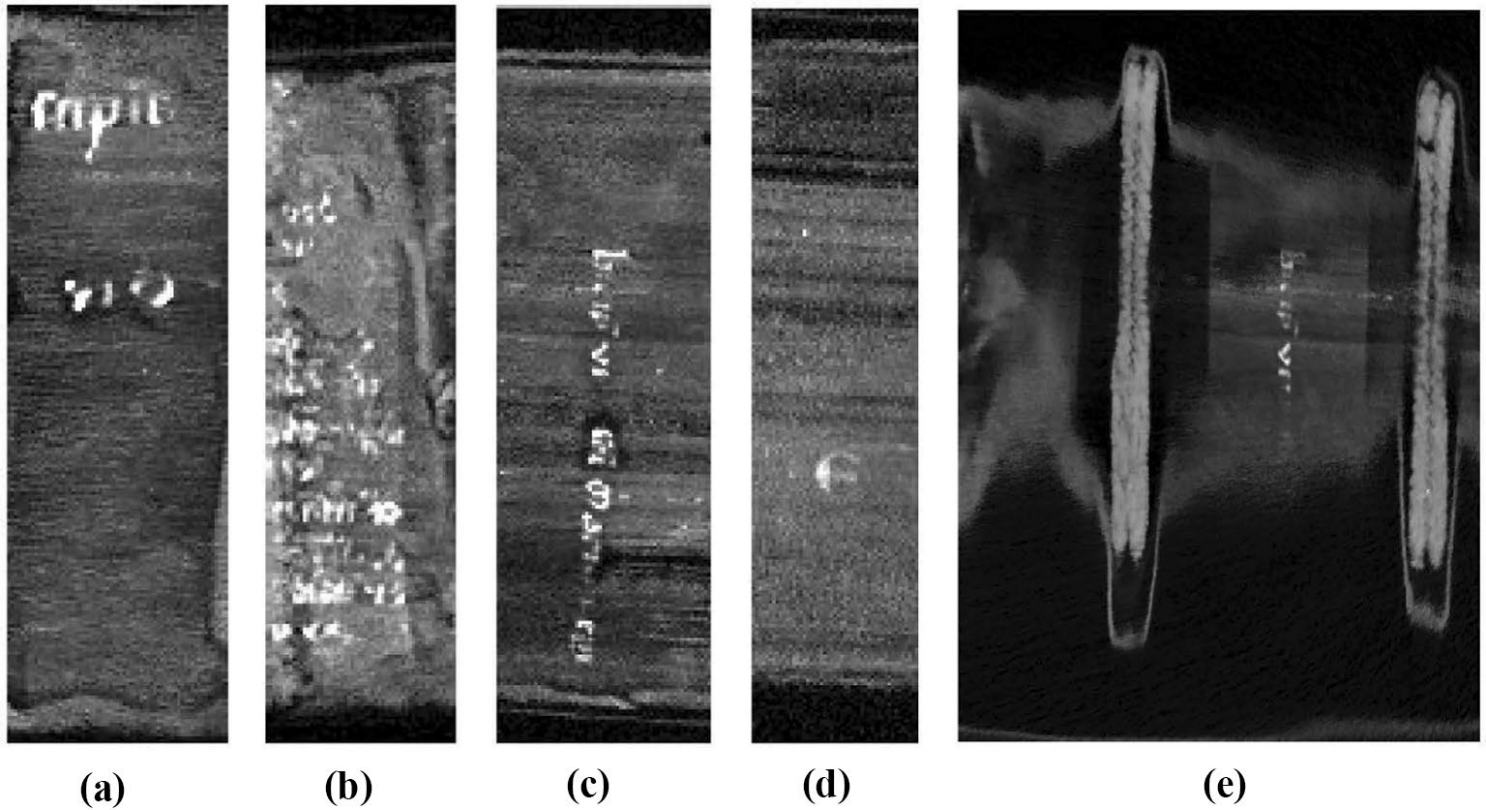
**a**-**d** LOGISMOS-flattened CT images of parchment with visible writing; **e** a single slice of the CT volume centered on the text that can be seen in the original CT volume prior to LOGISMOS-flattening. Note that while it is possible to determine presence of text in the original CT volume, LOGISMOS-flattening facilitates the visualization and reading of a much larger portion as shown in **c**

The acquired CT images, which were exported from the scanner as DICOM images, were analyzed using LOGISMOS (Layered Optimal Graph Image Segmentation for Multiple Objects and Surfaces) [39]**,** a comprehensive segmentation framework of algorithms that is widely used in the medical image processing community. The earliest publications on LOGISMOS [40, 41], which concerned its employment to segment multiple “terrain-like” 3D surfaces such as those associated with human retina layers imaged by OCT (Optical Coherence Tomography) motivated IISICCA’s decision to apply LOGISMOS to segment the curved tawed leather and parchment 3D surfaces. The 2D DICOM images from the scanner (each of which is acquired from a plane approximately parallel to the spine as shown in Fig. 4) were stacked based on their metadata to form a single 3D image and saved with NIfTI file format (which preserves the original 16-bit intensity resolution) for easy processing and visualization for LOGISMOS. The LOGISMOS 3D segmentation results are illustrated in Fig. 5, where three curved surfaces — those of the outer tawed leather cover, the parchment, and the spine — were segmented simultaneously. Because each of the segmented surfaces only intersects with a vertical column of pixels in Fig. 5 exactly once — an inherent property of LOGISMOS — and the surfaces have small curvatures, the parchment surface was virtually flattened by extracting voxels on the 3D surface to create 2D images of the parchment spine liners as shown in monochrome images in Figs. 6–8. Each of these 2D images contained the lining layer on a single plane.

Such flattening brought all relevant information into one 2D plane and thus made it possible to detect the existence of text on the parchment spine-lining layer beneath the tawed leather binding in all three volumes, including the control volume. Visible letters in both the Gothic script on the strips nearest the endbands and the earlier script of the transverse spine liners have thereby been detected, identified, and in some cases made legible.

As anticipated, our CT scans captured the structures of the fragments and their placement on the spine itself in all three volumes (Figs. 5, 6a-b). For example, the digital camera photograph in Fig. 6a is vertically bisected by the edge of a parchment strip adjacent to the stitched endband on the left; to the right of the first, this image shows a second strip bearing a light brown script at a 90° angle to the writing on the left. These features are detectable in the CT image, Fig. 6b. The latter also reveals (1) that the first strip has been folded under itself on the lefthand side, so that the endband stitches (their holes visible as a vertical row of black dots to the left) are supported by having to pass through a doubled parchment layer, and (2) that the second strip on the right continues under the first. A slice of the CT image, processed to segment the distinct layers of the covered spine (Fig. 5), visualizes the relationship of the parchment fragments on the spine to the outer tawed leather covering.

Comparison of the CT images with the exposed spine of the control sample confirms the partial detection by this method of writing that is visible to the naked eye, as well as that of additional letters that are not visible by visual observation alone. Through this comparison we have established that all the letters visible in our scans are rubrics or single red letters. In one example of visualized binding structure (Fig. 6a, b), faint letters emerge in the CT-obtained images precisely where we can see with the naked eye that red letters have been abraded. That our initial CT settings capture only red letters appears to confirm our hypothesis that the pigment used for them is vermilion, that is, the mineral cinnabar, mercury sulfide (HgS). This would accord with the MA-XRF results obtained by Duivenvoorden et al. as shown in their Fig. 3 [24]. Moreover, we can see some uppercase letters that are not visible by naked-eye observation of the control volume’s exposed spine (Fig. 7a, b). In this example, CT scanning brings into view both a clear majuscule (uppercase) ***F*** that is less easily seen in the photograph produced by a digital camera, and another large letter that is entirely invisible in the photograph. We believe this second large letter to be an initial on the reverse side of the manuscript fragment, in which case this letter (also reversed) is a form of lowercase uncial ***D*** (identifiable by the long horizontal rather than ascending stroke), typical of many Latin bookhands from the twelfth-century onward. These results led to the conclusion that the parchment linings in this volume (and perhaps the others in this series) likely contain writing on both sides, only one of which is exposed in the control sample.

**Fig. 9:**
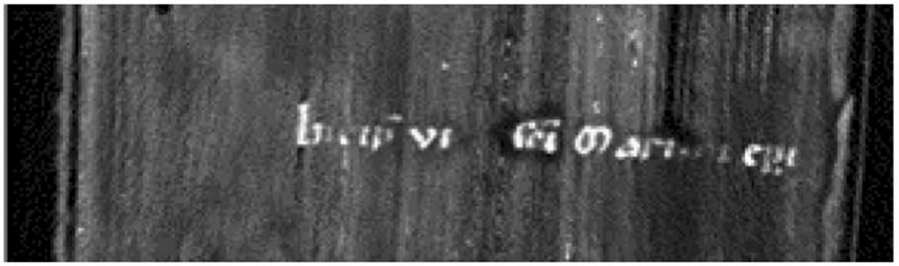
Detail of LOGISMOS-flattened image shown above in 8c turned sideways to follow normal Latin-text orientation of writing left to right. We used these and adjacent slices of the NIfTI images to﻿ reveal different portions of the legible rubric “Incip(it) vit[a] s(an)c(t)i Mar[t]ini e(pisco)pi” (see supplementary files)

Finally, CT images of the two non-control volumes yield letters (Fig. 8a-e), whole words — e.g. “sapi(enti)e/sapi(enti)a” in Fig. 8a — and at least once an entire rubricated phrase (Fig. 8c,e, 9), which we reconstruct as “Incip(it) vit[a] s(an)c(t)i Mar[t]ini e(pisco)pi” (where parentheses complete standard medieval Latin abbreviations and brackets supply letters that are not fully visible), i.e., “[here] begins the *Life* of St. Martin, bishop” of Tours, a popular saint’s life during the Middle Ages.

## Discussion

Given the limited detection of the writing that we know is present on the spine lining of one of the three volumes, namely our “control” volume, we suspect that the composition of the inks present on the samples differ in density level, and that the majority of the ink present is from a lower density source, which would require imaging at lower energy levels to be detected. Further study of these volumes is underway using additional medical imaging technology (photon-counting CT) that we expect to yield greater detection of writing on all three samples. While CT scanning technologies permit much faster acquisition of 3D data than the 2D data that mobile MA-XRF yielded for Duivenvoorden et al*.*, any evaluation of time savings must take into account the significant time required for data reconstruction to produce the DICOM images for analysis and processing.

## Conclusion

Over the last two decades scholars have deployed CT scanning technologies with varying degrees of success to recover virtually what Seales [26, 42] has felicitously described as the world’s “invisible library”— a library that would otherwise remain hidden within valuable, sometimes extremely damaged, cultural artifacts. IISICCA’s imaging experiments using CT scanning offer new avenues for rapidly discovering and in some cases making legible the remnants of medieval books hidden within tawed or leather bindings. The large gantry size (Fig. 4) and fast scan time of a medical DECT scanner makes scanning large numbers of manuscripts possible without customized equipment. Additionally, the dual energy capabilities of state-of-the-art scanners may allow for scan customization, targeting specific ink compositions. This will be further supported using recently emerging photon counting CT scanners which allow for the binning of photon energies into more than just two energy ranges [43].

We have confirmed that, unlike mobile MA-XRF scanning, CT scanning is a cost- and time-effective means for detecting medieval manuscript fragments beneath outer tawed or leather bindings. This makes CT scanning useful for screening collections of rare books to discover which ones contain fragments for investigation. Moreover, because the seemingly difficult problem of segmenting images of curved surfaces was solved by LOGISMOS fifteen years ago, its use to analyze the data obtained by the CT scanning of books promises to be successful in visualizing legible texts on the curved parchment fragment surfaces. At present, however, both MA-XRF scanning, despite its problems of focus, and IRT have resulted in more complete detection of inks and consequently more legible text on manuscript fragments in or under bindings in the Leiden and Rome samples *except* when scanning bindings made of tawed or leather skins. Given the common use of tawed and leather skins in early modern book bindings, further experimentation with DECT scanning of early modern book spines is warranted.

## Data Availability

3D NIfTI images that are the basis for those analyzed in this published article are available as supplementary files at: https://iisicca.uiowa.edu/resources.

## Footnotes

1 “Inuenias etiam disiecti membra poetae,” are the words of Horace, *Satires*, 1.4.62.

2 The Leiden research team also visualized the earlier, erased text of an important palimpsest in the same collection.

3 Using the naked eye and/or enlargement of a digital photograph, we can identify passages from 1 Kgs (1 Sam) 7:3–4 (but transcribing here the spelling of the manuscript, with italics indicating departures from the modern reconstruction of the medieval Vulgate): “[Ait autem Samuh]el ad univers*u*m domum Israel, dicens: Si in toto corde [vestro revertimini ad] Dominum, auferte deos alienos de medio vestri, Baa[lim et Astaroth: et] preparate corda vestra Domino, et servite ei soli, et eruet [vos de manu Philisthiim. Abstulerunt] ergo filii Israel Baalim…” 1 Kgs 7:17–8:3: “[Revertebaturque] in Ramatha: ibi enim erat domus ejus, et ibi [judicabat Israelem: aedificavit] etiam [ibi] altare Domino. Factum est autem cum senuisset Samuhel, posuit filios suos judices Israel. Fuit[que nomen filii ejus] primogeniti Joel: et nomen secundi [Abia, judicum in Bersabee. Et] non ambulaverunt filii illius in viis ejus;” 1 Kgs 8:6–8: “ad Dominum. Dixit*que* Dominus ad Samuhelem: [Audi vocem populi in om]nibus quae locuntur tibi: non enim t[e abiecerunt, sed] me, ne regnem super eos. Juxta omnia opera sua, [que fecerunt a die qua] eduxi eos de Aegypto usque ad diem hanc. Sic[ut dereliquerunt me, et] servierunt diis alienis, sic faciunt etiam tibi.”.
